# Enhancing the Nutritional and Functional Properties of *Auricularia auricula* through the Exploitation of Walnut Branch Waste

**DOI:** 10.3390/foods11203242

**Published:** 2022-10-17

**Authors:** Zhenkun Hao, Wen’e Zhang, Fenghua Tian, Rong Wei, Xuejun Pan

**Affiliations:** 1 Engineering Research Center for Fruit Crops of Guizhou Province, Guiyang 550025, China; 2 College of Agriculture, Guizhou University, Guiyang 550025, China

**Keywords:** walnut sawdust, black agaric, growth rate, substitute substrate, extracellular enzyme activity, comprehensive evaluation

## Abstract

As the third most edible fungus in the world, *Auricularia auricular* needs a lot of sawdust for cultivation; thus, it is a win–win method to develop waste wood sawdust suitable for black agaric cultivation. This study evaluated the growth, agronomic characters and nutritional quality of *A. auricula* cultured on different ratios of miscellaneous sawdust and walnut waste wood sawdust, and comprehensively analyzed the feasibility of cultivating black agaric with walnut sawdust using principal component method (PCA). The results showed that the macro mineral elements and phenolic substances in walnut sawdust were significantly higher than those of miscellaneous sawdust by 18.32–89.00%. The overall activity of extracellular enzymes reached the highest when the ratio of the substrate was 0:4 (miscellaneous sawdust: walnut sawdust). The mycelia of 1:3 substrates grew well and fast. In addition, the growth cycle for *A. auricula* was significantly lower for 0:4 (116 d) than for 4:0 (126 d). Then, the single bag yield and biological efficiency (BE) were highest at 1:3. Moreover, the nutrients and mineral elements of *A. auricula* cultivated in walnut sawdust were significantly higher than that of miscellaneous sawdust, expect for total sugar and protein, and the highest overall value was found at 1:3. Finally, the results of comprehensive evaluation by PCA showed that the D value was the highest when the substrate was 1:3 and the lowest when the substrate was 4:0. Therefore, the substrate ratio of 1:3 was the most suitable for the growth of *A. auricula*. In this study, the high yield and quality of *A. auricula* were cultivated by waste walnut sawdust, which provided a new way to utilize walnut sawdust.

## 1. Introduction

Black agaric (*Auricularia auricula*), as the third most important cultivated edible fungus in the world, is widely cultivated in Asian countries such as China and Japan due to its unique cooking style [1,2,3]. In recent years, scientific evidence has demonstrated its potential value in functional foods and medicines [4], and as a result, it is widely used in medicinal materials, fermented food, antibiotics, and oxidants, etc. [5]. In China, *A. auricula* is very popular among consumers for its high protein, low fat, and rich functional nutrients such as polysaccharides, polyphenols and flavonoids [6]. Currently, with improving people’s living standards and pursuing a healthy lifestyle, the consumption demand for *A. auricula* is increasing continuously. In 2019, China’s annual production of *A. auricula* reached 7.018 million tons, making it the second largest cultivated edible fungus [7]. However, with the continuous expansion of the cultivation area of *A. auricula,* which leads to the overexploitation of sawdust in many regions, the price of sawdust used for the cultivation of *A. auricula* keeps rising, which not only leads to increases in the *A. auricula* cultivation cost, but also makes the contradiction between edible fungi and forest increasingly prominent [8]. Therefore, to protect the ecological environment and achieve sustainable production, it is timely and necessary to develop alternative cultivation techniques for *A. auricula* using other waste sawdust chips as substrate.

As one of the four major dried fruits in the world, walnut is a vital woody oil tree species in China, with its planting area and yield ranking first in the world [9]. In recent years, with the further improvement of the development quality of the walnut industry in China, the degree of walnut cultivation and standardization has been continuously improved. In 2018, the cultivation area of walnuts in China reached 7.33 million hectares, with approximately 22 million tons of sawdust residues generated annually in walnut producing areas through variety improvement, density adjustment, plastic pruning and trimming (http://www.crnews.net/) (accessed on 4 October 2020). At present, walnut waste sawdust is mainly solved by traditional ways such as stacking and burning, which not only leads to a large amount of waste of walnut nutrition but also pollutes the air environment and environmental aesthetics [10]. How to utilize this considerable biomass resource reasonably and effectively has become an important issue that needs to be solved urgently.

Studies have found that walnut sawdust contains a large number of organic compounds such as lignin, cellulose, and hemicellulose [11]. The wood-rotting fungus can secrete extracellular enzymes to decompose lignin and cellulose biopolymers and promote growth, which is a highly efficient biodegrading [12,13]. The prospects for utilizing these large undeveloped materials are excellent, primarily if achieved through a combination of environmentally sound management and the generation of new value-added products. An indicative case for fulfilling this prerequisite is the controlled solid-state fermentation of various plant residues to produce edible mushrooms [14,15,16,17]. Previous studies have found that wood-rotting fungus showed different lignocellulosic degradation abilities on different substrates, which makes the growth rate of mycelia, biomass yield, and extracellular enzyme activity greatly influenced by the properties and composition of lignocellulosic substrates [18]. Sun et al. [19] found that phenols contained in walnuts could inhibit fungal growth at a particular concentration. However, Maria et al. [20] found that the culture of *Ganoderma lucidum* with hickory lignocellulose waste as solid substrate not only significantly increased yield, but also effectively alleviated environmental problems. The previous results showed that *A. auricula* could secrete a large number of extracellular enzymes to decompose and utilize biological macromolecules in walnut sawdust [21]. However, the research on edible fungi cultivated by waste materials has focused on the matrix, and less research has been conducted on the nutrient composition and bioactive compound content of basidiocarp. In addition, it was found that the biological efficiency of edible fungi was closely related to each substrate’s nutrient element content and cellulose content. Finally, there are a little data available on the potential uses of waste walnut sawdust in the cultivation of edible fungi. Thus, the purposes of this study were (1) to assess the effect of different content of walnut sawdust on extracellular enzyme activity, basidiocarp yield and nutrient composition of *A. auricula*; and (2) through comprehensive comparison, to further explore the biodegradation ability of walnut sawdust and construction of walnut cultivation *A. auricula* technical scheme according to the results of (1). This study not only provides a solution for the reuse of walnut sawdust in walnut producing areas in China, and effectively reduces environmental pollution, but also builds a high-quality and low-cost production system of *A. auricula*.

## 2. Materials and Methods

### 2.1. Materials and Chemicals

For this study, a commercial strain of *A. auricula* was used; the fungus is routinely maintained in potato dextrose agar (PDA) at 4 °C and preserved in the culture collection of the institute of Edible Fungi, Guizhou University (Guiyang, China), under the accession code ‘2020062301’. The fungal strains were activated on a PDA medium at 24 °C for subsequent experiments.

To prepare the mushroom cultivation substrates, walnut sawdust, miscellaneous sawdust, wheat bran, lime and gypsum were provided by the Fenghe Agricultural Company (Guiyang, China).

The chemicals used to determine of physiological indicators, such as glucose, peptone, MgSO_4_ and KH_2_PO_4_, were provided by Kaixin biotechnology company (Guiyang, China).

### 2.2. Fungal Mycelium Growth Media

The culture substrate was thoroughly and evenly mixed. The water content was controlled at 60% using a soil moisture meter (Spectrum, Lincolnwood, IL, USA). The substrate was evenly spread into a glass petri dish with a diameter of 9 cm. After 121 °C autoclaved (Zealway, Xiamen, China) for 1 h, it was cooled to room temperature. Then, a 5 mm hole punch was used under aseptic conditions to drill holes evenly along the edge of the PDA plate covered with mycelia, and the mycelium blocks were inoculated into the sterilized matrix. The growth rate of mycelia was measured on the 10th day of culture.

### 2.3. The Preparation of Liquid Strains and Mushroom Cultivation Process

Liquid media consisted of potato (100 g/L), wheat bran (40 g/L), glucose (20 g/L), peptone (2 g/L), brown sugar (15 g/L), MgSO_4_ (1 g/L), KH_2_PO_4_ (2 g/L). The 5 mm diameter of the activated mycelium was collected for the preparation of liquid strains. Liquid spawn was prepared by culturing mycelial plugs (5 mm in diameter, 6 plugs/flask) in 1 L flasks containing 400 mL of liquid media, and shook in a shaker (Bluepard, Shanghai, China) at 160 rpm at 24 °C for 8 days in darkness [22].

In this study, walnut sawdust was used to replace the miscellaneous in the basic formula (miscellaneous 78%, wheat bran 20%, lime 1%, gypsum 1%), and the proportion of other excipients remained unchanged. These raw materials were used to prepare the following five substrates: (a) miscellaneous sawdust: walnut sawdust 4:0 (control); (b) miscellaneous sawdust: walnut sawdust 3:1; (c) miscellaneous sawdust: walnut sawdust 2:2; (d) miscellaneous sawdust: walnut sawdust 1:3; and € miscellaneous sawdust: walnut sawdust 0:4. After the cultivation substrate was fully mixed according to each formula, distilled water was added to adjust the water content of the medium, and the water content was controlled to 60% with a soil moisture meter (Spectrum, Lincolnwood, IL, USA). Then, the cultivation substrate was evenly loaded into edible fungus culture bags, and the weight of each bag was 700 g. The filled bags were then autoclaved (Zealway, Shanghai, China) at 121 °C for 2 h, and cooled to below 20 °C. To ensure the same number of spores were inoculated in each bag, the inoculum of liquid strains was strictly controlled at 15 mL. After inoculation, all bags were incubated in the dark at 24 °C [23], after its mycelia filled the bag, each formulation was cultivated for twenty additional days to ensure physiological maturity. Each bag was then pierced with a perforator (Qianzhenyuan, Heilongjiang, China) and transferred to a cultivation room. All treatments were maintained in the same room under the same conditions (22 °C, 90–95% relative humidity, keep ventilation) to facilitate basidiocarp growth [24]. Once the fructification started, fully frown fruiting bodies in the mushroom production were harvested.

### 2.4. Determination of Nutrient Elements in Walnut and Miscellaneous Sawdust

The collected miscellaneous sawdust and walnut sawdust were dried at 50 °C in a blast drying box, and then crushed through a 40-mesh screen for later use. The carbon content in sawdust was measured by the sulfuric acid–potassium dichromate oxidation method [25]. The content of nitrogen in sawdust was determined by the Kjeldahl method [26]. According to the agricultural industry standard NY/T 1653-2008 of the People’s Republic of China, the content of mineral elements in sawdust chips was determined by inductively coupled plasma emission spectrometry [27].

### 2.5. Determination of Extracellular Enzyme Activity

The substrates covered by *A. auricula* mycelia during mycelia growth and basidiocarp formation were collected. The substrate covered by mycelia was weighed at 2 g and properly ground, then transferred to a 50 mL centrifuge tube filled with 40 mL distilled water, and incubated (Thermo, Waltham, MA, USA) at 37 °C for 2 h. After filtration with 4 layers of gauze, the filtrate was kept at a constant volume of 50 mL and centrifuged (Thermo, Waltham, MA, USA) at 4 °C and 8000 rpm for 10 min. The obtained supernatant was stored (Thermo, Waltham, MA, USA) at −80 °C for the subsequent determination of extracellular enzyme activity of *A. auricula* [28].

#### 2.5.1. Determination of Laccase Activity

Laccase activity was measured using the laccase assay kit (Sorlabio, Beijing, China). The amount of enzyme required to oxidize 1 nmoL of substrate ABTS per minute per gram of sample was one unit of enzyme activity [29]. Then, 0.1 g of tissue was weighed and added to 1 mL of extract for homogenization in an ice bath. Then, centrifuged (Thermo, Waltham, MA, USA) at 10,000× *g* 4 °C for 10 min, the supernatant was placed on ice. The agent to be tested was added in a 1 mL glass cuvette, thoroughly mixed, and then, the absorbance value was measured at A1 at 420 nm for 10 s, quickly placed in a 45 °C water bath (Ahyq, Changzhou, China) for 3 min, and quickly wiped dry to measure the absorbance value A2 at 190 s (Metash, Shanghai, China).

The laccase activity (U/g) = ΔA ÷ ε ÷ D × Vt × 109 ÷ V ÷ T

ε: ABTS molar extinction coefficient (36,000 L/mol/cm); D: cuvette diameter (1 cm); Vt: total reaction volume (0.001 L); V: sample volume in reaction (0.15 mL); T: reaction time (3 min).

#### 2.5.2. Determination of Polyphenol Oxidase Activity

Polyphenol oxidase activity was determined using catechol as substrate. The reaction mixture was 0.5 mL enzyme extracting solution, 5 mL phosphate buffer (0.2 mol/L; pH 6.0), and 1 mL catechol solution (0.12 mol/L). The reaction solution was soaked (Ahyq, Changzhou, China) at 37 °C for 15 min and then quickly placed in ice water. The reaction was terminated by standing for 3 min, and then, the absorbance value was measured at 410 nm (Metash, Shanghai, China). Polyphenol oxidase activity was expressed in units per gram (U/g) and was defined as a change of absorbance value of 0.001/min per gram of sample as one enzyme activity unit [30].

#### 2.5.3. Determination of Cellulase Activity

Cellulase activity was determined with 15 mg/mL sodium carboxymethylcellulose in sodium acetate buffer (0.1 mol/L, pH 5.5) as substrate. Subsequently, 2 mL sodium carboxymethyl cellulose solution was mixed with 1 mL extract and incubated at 37 °C for 30 min. After adding 0.5 mL DNS reagent and vortexed for 3 s, the reaction was terminated by placing it in boiling water for 5 min. The absorbance was subsequently measured at 540 nm, and the concentration was calculated according to the formula. Cellulase activity was expressed in units per gram (U/g), which was defined as the amount of enzyme required to release 1 μmoL of reducing sugar from the degradation of sodium carboxymethyl cellulose solution per minute [31].

#### 2.5.4. Determination of Xylanase Activity

Xylanase activity was determined with 100 mg/mL xylan in sodium acetate buffer (0.1 mol/L, pH 5.5) as substrate. The xylan solution (2 mL) was mixed with 1 mL extracting solution and incubated at 37 °C for 30 min. Then, 0.5 mL of DNS reagent was added, and after vortexing for 3 s, the reaction was terminated by placing it in a boiling water bath for 5 min. Absorbance was measured at 540 nm. Xylanase activity was expressed in units per gram (U/g) and was defined as the amount of enzyme required to release 1 μmoL of reducing sugar per minute.

### 2.6. The Growth Evaluation of A. auricula

To order to evaluate the effects of different cultivation substrates on each growth stage of *A. auricula*, the following parameters were studied in this study [32]: (a) mycelial growth rate; (b) bag-filling time; (c) the number of days of primordia formation and (d) days of basidiocarp maturation.

Average mycelium growth rate (mm/d) = colony radius/culture days;

Bag full time (d) = the date when 90% of the culture bags was filled with mycelia—the date when the liquid strains were inoculated;

Primordia formation days (d) = date of primordia formation on 90% of culture bags—date of physiological maturity;

Days of basidiocarp maturation (d) = date when 90% of the basidiocarp diameter exceeds 4 cm—date when the basidiocarp begins to grow.

### 2.7. Determination of Production and Cultivation Benefit of A. auricula

To evaluate the effects of different walnut sawdust supplemental ratios on the production efficiency of *A. auricula*, this study measured: (a) single bag yield; (b) soaking rate and (c) biological efficiency. Ten bacteria bags were randomly selected for each treatment, and the average value was taken (3 replicates).

Yield per bag (g) was the sum of the dry weight of three batches of black fungus harvested in a single belt.

Soaking rate (%) = weight of dried agaric after soaking/dry weight of black agaric × 100%

Biological efficiency (%) = dry weight of each bag of black fungus/dry weight of corresponding matrix × 100%

### 2.8. Determination of Nutrient Elements in Basidiocarp of A. auricula

Before chemical analyses and unless otherwise stated, mushroom samples were dried to constant weight by maintaining them at 60 °C for 48 h and were then converted to powder. Moisture content was determined according to the direct drying method of the China National Food Standard GB 5009.3-2016. Crude protein was calculated from total nitrogen content (respective measurements were verified by the macro Kjeldahl method using a Kjeltec analyzer unit; Foss Tecator AB, Hoganas, Sweden) by employing the converting factor 4.38 [33], instead of the commonly employed 6.25 since mushrooms contain significant amounts of non-protein N-rich organic compounds (e.g., mainly chitin). Crude fat content was determined according to the Soxhlet extraction method by the China National Food Standard GB/T 15674-2009. Polysaccharide content was determined according to the method by Song et al. [34]. Total polyphenol content was performed according to the method by Wang et al. [35]. Total flavonoid content was determined using the method by Song [34]. Mineral element content was determined using the inductively coupled plasma emission spectroscopy (Shimadzu, Hong Kong, China) based on the method by the Agricultural Industry Standards of the People’s Republic of China NY/T 1653-2008.

### 2.9. Comprehensive Evaluation of A. auricula Cultivated in Different Walnut Sawdust Ratio

To comprehensively compare the effects of different walnut sawdust supplemental ratios on *A. auricula* cultivation, the data were analyzed by PCA referring to the method of Wang et al. [36]. Excel 2013 was used for data collation and statistics; SPSS 26.0 software was used for correlation analysis and PCA. Firstly, the membership function method was performed to conduct PCA on the data, and the comprehensive index values were valued. Secondly, the membership function values of all comprehensive indicator indexes were calculated. Next, the weight and evaluation were calculated according to the contribution rate of the comprehensive index. Finally, the optimum amount of walnut sawdust for the cultivation of *A. auricula* was determined [37].

### 2.10. Statistical Treatment of Experimental Data

Excel software was used for statistical data, and SPSS 26.0 software was used for one-way analysis of variance. All the assays followed a completely random design with three replications. The results were evaluated using analysis of variance (ANOVA), and significant differences among arithmetic means were determined by the LSD test at 5% probability. Spearman’s correlation coefficient was used to assess the relationship between extracellular enzyme activity and basidiocarp yield of the variable *A. auricula* (*p* < 0.05).

## 3. Results and Discussion

### 3.1. Comparison of Nutritional Components of Walnut and Miscellaneous Sawdust

**Figure 1:**
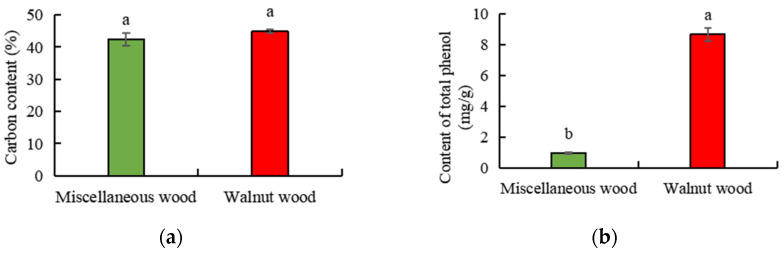
Comparison of (**a**) carbon content and (**b**) total phenol content of walnut and miscellaneous sawdust. Different lowercase letters within each column are significantly different at *p* < 0.05 between treatments.

The selection of suitable substrate material is the key to the successful production of edible fungi. As shown in Figure 1, it was found that the carbon content showed no significant difference (*p* < 0.05) between walnut sawdust and miscellaneous sawdust (Figure 1a), while the total phenolic content (Figure 1b) and the content of major mineral elements (Table 1) of walnut sawdust were significantly higher (*p* < 0.05) than that of miscellaneous sawdust. Further analysis showed that the content of phenols in walnut sawdust was 89.00% higher than that in miscellaneous sawdust. Moreover, among the major mineral elements, the contents of N, P, K, Ca and Mg in walnut sawdust were 18.32%, 72.63%, 44.80%, 35.69% and 33.33% higher than those in miscellaneous sawdust, respectively. However, as for the micronutrient’s mineral, the contents of Fe, Zn and B in walnut sawdust were significantly lower (*p* < 0.05) by 15.24%, 60.76% and 12.58% than those in miscellaneous sawdust, while the contents of Cu and Mn in walnut sawdust were significantly higher (*p* < 0.05) by 41.76% and 5.88% compared to those in miscellaneous sawdust (Table 1). This was because compared with other fruit trees, walnut production had high demand for mineral nutrients, especially a large number of elements [38]. It was found that walnuts absorbed 4~6 times more N from the soil than Citrus reticulata (*Citrus limon*), and lemon (*Armeniaca vulgaris*) with the same conversion yield [39]. For example, Srivastava et al. [40] compared the nutrient absorbed by 22 major fruit crops and found that under the same yield, the total amount of N, P and K absorbed from walnut ranked fourth, which was 1.2~6.7 times that of other fruit crops. Previous studies found that C and N sources were two essential factors affecting the composition and activity of critical enzymes of lignin degradation by fungi [41,42,43]. In addition, suitable matrix metal ions had a significant impact on the secretion of nutrients and functional active components by extracellular enzymes of edible fungi mycelium growth. For example, Song et al. [34] found that adding Mn and Ca to the liquid medium of *Sanghuangporus* could effectively improve the yield of acidic polysaccharide. Adil et al. [44] found that metal ions inhibited the hyphal growth of Lentinus edodes but promoted the production of polysaccharides. Therefore, it was determined that walnut could be used as raw material for A. auricula cultivation by comprehensive analysis of the nutrient element contents of two kinds of sawdust.

### 3.2. The Effect of Walnut Sawdust Ratio on the Extracellular Enzyme Activities

**Figure 2:**
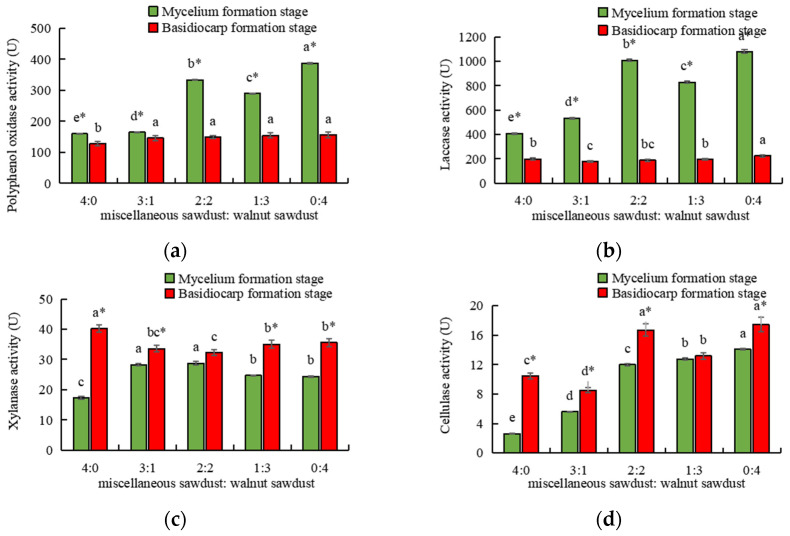
Effects of different walnut supplemental levels on extracellular enzyme activity of (**a**) polyphenol oxidase; (**b**) laccase; (**c**) xylanase; (**d**) cellulase during mycelium and basidiocarp formation stage. Different lowercase letters within each column are significantly different at *p* < 0.05 between treatments. * is significantly different at *p* < 0.05 between the formation stage.

Previous studies had found that edible fungi could secrete a variety of extracellular enzymes during growth, causing natural polymers such as lignocellulose, proteins, and nucleic acids in the plant tissue to be broken down into small molecules that facilitate the uptake of mycelia and basidiocarp, thus providing nutrition for the growth of edible fungi hyphae, primordium formation and basidiocarp growth and development [45,46,47]. As shown in Figure 2a,b, in both mycelia formation stages (MFS) and basidiocarp formation stages (BFS) of *A. auricula*, the activities of polyphenol oxidase and laccase of walnut sawdust were significantly higher (*p* < 0.05) than those of miscellaneous sawdust. In the MFS, the activities of polyphenol oxidase and laccase were significantly different (*p* < 0.05) among different walnut sawdust addition ratios, and the activities of polyphenol oxidase and laccase were the highest when the walnut sawdust and miscellaneous sawdust ratio was 0:4. However, there was no significant difference in the activities of polyphenol oxidase and laccase in different walnut supplemental levels during BFS. As shown in Figure 2c,d, during the BFS of *A. auricula*, the activities of xylanase with walnut supplemental were significantly lower (*p* < 0.05) than those of miscellaneous sawdust, while in the MFS, the activities of xylanase and cellulase were higher with walnut supplemental. Regardless of the growth stage or the type of extracellular enzymes, the activities of four extracellular enzymes in *A. auricula* were always in the leading position when the walnut sawdust and miscellaneous sawdust ratio was 0:4.

It was found that polyphenol oxidase and laccase were the main enzymes degrading lignin. As the molecules of laccase and polyphenol oxidase contain copper atoms, they play an electron transport role during the oxidation of substrates catalyzed by the enzyme. They participate in catalysis by strengthening the reaction orientation or reversibly changing the oxidation state of the enzyme and substrate to regulate the redox reaction, thereby stabilizing the enzyme structure and activating the enzyme activity [48,49,50]. Yang et al. [51] found that adding Cu could improve the laccase activity of *pleurotusostreatus* to a certain extent and promote the differentiation of primordia and the development of fruiting bodies. Zhu et al. [52] found that adding Cu to the culture medium could enhance the laccase activity of *oyster mushrooms*. According to Table 1, the copper content in walnut sawdust was significantly higher (*p* < 0.05) than that of miscellaneous sawdust. It was hypothesized that Cu in walnut sawdust played a greater role in the electron transfer during the substrate oxidation catalyzed by polyphenol oxidase and laccase than that of miscellaneous sawdust, and could stabilize the enzyme structure more, so that the activities of polyphenol oxidase and laccase were significantly increased compared with miscellaneous sawdust [48,50].

In addition, it was found that laccase activity in MFS was significantly higher (*p* < 0.05) than that in BFS, and the laccase activity directly reflected the degradation capacity of lignocellulose by *A. auricula* [53]. It was hypothesized that laccase decomposes lignin in a large amount in the MFS, which fully exposed cellulose and hemicellulose, creating conditions for the massive degradation of cellulose and hemicellulose in the BFS. Xu et al. [54] found that during the growth and development of *lentinus edodes*, the changing trend of laccase activity was similar to that of lignin decomposition in cultivated materials. He et al. [55] found that the lignin content of grape sawdust was lower than that of oak sawdust, which may lead to the lower lignin enzyme activity of each grape sawdust formula than the control. In conclusion, the extracellular enzyme activity can reflect the degradation characteristics of cultivated materials to a certain extent. At the same time, the accumulation of lignin products and the reduction of the C/N ratio in the medium inhibited the laccase activity at the BFS, resulting in the rapid decline of laccase activity. Even so, the laccase activity secreted by mycelia of *A. auricula* in the culture material was still higher than the other three enzymes, and the maximum laccase activity of MFS was 2.78 times that of polyphenol oxidase, 38.50 times that of xylanase and 76.51 times that of cellulase, indicating that laccase dominated the degradation of lignin in the medium by mycelia of *A. auricula*. This was consistent with the results of the study of extracellular enzyme activity of *Hypsizygusmarmoreus* by Wu et al. [51].

### 3.3. Effect of Walnut Sawdust Addition Ratio on the Growth Cycle of A. auricula

**Figure 3:**
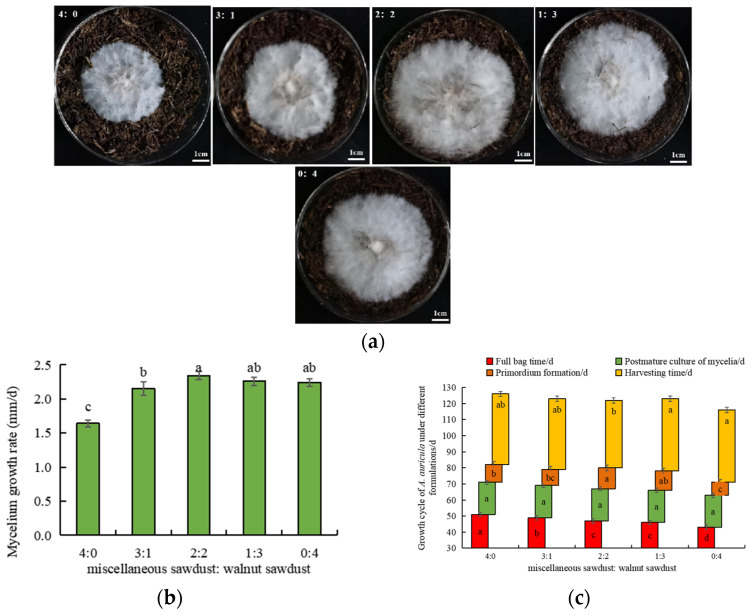
(**a**) Mycelia growth status, (**b**) mycelia growth rate and (**c**) growth cycle of *A. auricula* on substrate ratio was 4:0; 3:1; 2:2; 1:3 and 0:4. Different lowercase letters within each column are significantly different at *p* < 0.05 between treatments.

As one of the important indexes of the growth characteristics of edible fungi, the growth rate is often used as an important basis for the selecting cultivation substrates to produce edible fungi. Sun et al. [19] found that the content of polyphenols in walnut sawdust was higher than that in miscellaneous sawdust, and phenols significantly inhibited fungal growth at a certain concentration. Zhao et al. [56] found that the growth rate of mycelium was positively correlated with the activity of lignocellulolytic enzymes induced in *Pleurotuseryngii* DC. Gillet was cultured with a corncob, corn stover, wood chips and peanut shells as the culture substrates. Compared with miscellaneous sawdust, it was found that the mycelia with walnut sawdust supplement had more substantial growth potential, white and dense, and neat mycelia edges (Figure 3a).

Moreover, the mycelia growth rate of *A. auricula* with walnut sawdust was significantly higher than that with miscellaneous sawdust, and with the increase in walnut sawdust addition, the growth rate of *A. auricula* mycelia first increased and then decreased (Figure 3b). When the substrate ratio was 2:2 and 1:3, the mycelial growth amplitude was better than that of other treatments. It was found hypothesized that one of the reasons was that the addition of walnut sawdust increased the activity of polyphenol oxidase, which could oxidize phenols in walnut sawdust to quinone and then polymerize to melanin, thus preventing the inhibition of phenols on mycelia growth [35]. However, when the amount of walnut sawdust was too high, polyphenol oxidase could not remove phenolic substances in time, leading to a decrease in mycelium growth rate. Another reason was that in MFS, the activities of polyphenol oxidase, laccase and cellulase at substrate ratios of 2:2, 1:3 and 0:4 were significantly higher (*p* < 0.05) than those in other treatments, which aggravated the decomposition of lignocellulose and released more carbon sources, thus promoting the growth of mycelia. Maria et al. [20] confirmed the importance of the formulation for mushroom yield, finding that biological efficiency, yield, and dry BE were significantly increased in substrates supplemented with 100% hickory chips. It was further confirmed that the high similarity between the substrate formulation and the natural environment mushrooms growth may be the key to successfully obtaining high biological efficiency values.

Figure 3c showed the effects of walnut sawdust supplements on the growth cycle of *A. auricula*. It was found that with the increase in walnut sawdust supplement level, the time of mycelia full bag shortened, the time of primordia formation increased first and then decreased, while the basidiocarp growth time of *A. auricula* was the opposite. It was found that walnut sawdust could enhance the extracellular enzyme activities of MFS and BFS, facilitate the decomposition of cellulose, hemicellulose and lignin, and provide nutrients for basidiocarp formation [28]. Moreover, *A. auricula* only needed 116 days to be harvested when the substrate ratio was 0:4, which was much lower than that of miscellaneous sawdust culture (126 days) and other walnut addition formulas. The results showed that the walnut sawdust supplement could significantly shorten the cultivation period of *A. auricula*.

### 3.4. Effects of Walnut Sawdust Supplemental Ratio on Basidiocarp Agronomic Traits

**Figure 4:**
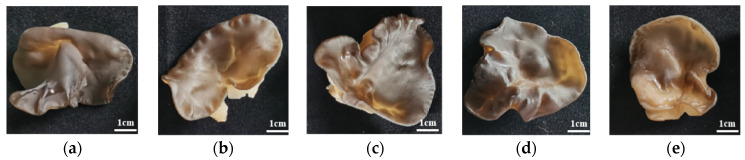

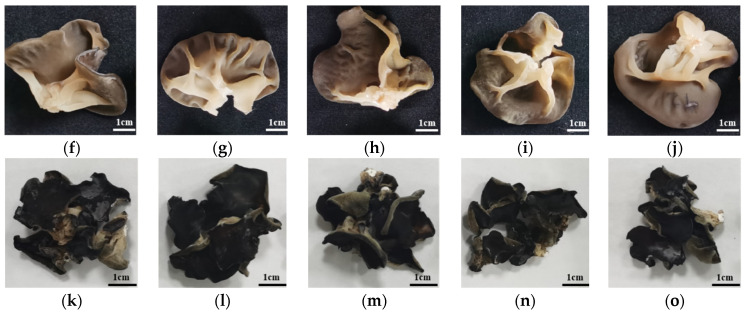
The ratio of miscellaneous sawdust and walnut sawdust was 4:0, 3:1, 2:2, 1:3, and 0:4 of *A. auricula* on the fresh front (**a**–**e**); fresh back (**f**–**j**) and dried sample (**k**–**o**).

**Figure 5:**
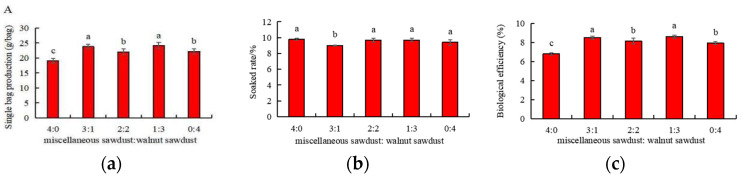
Effects of different proportions of walnut sawdust on single bag production (**a**), soaked rate (**b**) and biological efficiency (**c**) of *A. auricula* basidiocarp. Different lowercase letters within each column are significantly different at *p* < 0.05 between treatments.

As be seen from Figure 4, there was no significant difference in the growth status of *A. auricula* with different walnut supplemental levels, which indicated that *A. auricula* conducive to walnut cultivation was consistent with regular cultivation in terms of appearance and morphology, without variation. It further proved the feasibility of walnut sawdust cultivation of *A. auricula*. Studies on extracellular enzymes and cultivation characteristics of edible fungi showed that the activities of cellulase and hemicellulose of *A. auricula* were related to the degradation rate of cultivation substrate and the yield of basidiocarp [57]. Carboxymethyl cellulase activity in extracellular hyphae of *Pleurotusostreatus* and *Flammulinavelutipes* was positively correlated with basidiocarp yield [58]. It can be seen from Figure 5 that the single bag production and BE of *A. auricula* cultivated with walnut were significantly higher (*p* < 0.05) than that of miscellaneous, while the soaked rate increased first and then decreased with the increase in the proportion of walnut added. It was hypothesized that since the polyphenol oxidase and laccase activities of significantly higher (*p* < 0.05) than that of miscellaneous sawdust, polyphenol oxidase, and laccase were mainly related to the degradation of macromolecular material such as lignin, which accelerated the decomposition of aromatic macromolecule compound lignin [59] and provided rich nutrition with strains.

Further analysis showed that when the substrate ratio was 1:3, the single bag yield and soaked rate were the highest. By analyzing the laccase and polyphenol oxidase activity and yield, it was found that when the substrate ratio was 1:3, the activity of laccase and polyphenol oxidase was higher, and the yield of *A. auricula* was also higher. However, when the substrate ratio was 4:0, the enzyme activity was lower and the yield was relatively low. When the substrate ratio was 0:4, the enzyme activity kept at a high level, the mycelia grew fast, and the cultivation period was fast, but the yield was relatively low. The reasons may be that excessive enzyme secretion caused excessive consumption of matrix nutrients, and the carbon and nitrogen ratio imbalance was caused by excessive consumption of nutrients in cultivation and production, resulting in the decline of basidiocarp yield. The results showed that the extracellular enzyme activity of *A. auricula* was positively correlated with the basidiocarp yield, which was consistent with the results of Han et al. [45]. In addition, Zhao et al. [56] also found that the activity of extracellular enzymes directly affects the biodegradation ability.

### 3.5. Effects of Walnut Sawdust Added Ratio on Basidiocarp Nutrients and Mineral Nutrition

In this study, one important purpose of waste walnut wood was to produce nutritious and healthy edible fungi. The nutritional qualities and mineral nutrition of *A. auricula* with different formulations were further analyzed (Table 2 and Table 3). Table 2 showed that in addition to total sugar, adding walnut sawdust into the cultivation matrix could promote the contents of protein, fat, polysaccharide, total phenols and total flavonoids of *A. auricula*, and the promotion degree varies with the change of walnut sawdust supplement level. With the increase in supplemental walnut level, the total sugar content decreased significantly (*p* < 0.05), while the protein content did not change significantly. The contents of fat, polysaccharide, total phenol and total flavonoids increased first and then decreased with the increase in supplemental walnut level. Fat and polysaccharides decreased at 1:3, while total phenols and flavonoids decreased at 2:2. Generally, the nutritional quality of *A. auricula* when the substrate ratio was 2:2 and 1:3 was better than other treatments. Consistent with Table 2, the mineral element content of *A. auricula* with walnut addition was significantly higher (*p* < 0.05) than that with miscellaneous sawdust (Table 3). The content of Mg increased gradually with the increase in walnut sawdust supplemental level, while the other mineral elements increased first and then decreased with the increase in walnut sawdust supplemental level. Except for Zn, the mineral content of *A. auricula* was generally higher than that of other treatments when the substrate ratio was 1:3, indicating that the growth matrix was one of the factors affecting the quality of edible fungi (Table 3).

In recent years, phenolic compounds, polysaccharides, proteins, fibers and other bioactive molecules have attracted much attention due to their health benefits [38]. Previous studies showed that adding different kinds of sawdust to the cultivation substrate could improve the nutrient composition of mushrooms. For example, it was found that the contents of triterpenoid acid, free amino acid, and lovastatin in the basidiocarp formation stage of *Tricholomaulmus* were significantly increased by grape pomace plus wheat straw [60], while the β-glucan content in *Schizophyllumcommne* Fr. was significantly increased by the medium prepared by 94% grape pomace plus wheat straw [61]. Consistent with previous studies, it was found that the *A. auricula* total phenolic content of walnut sawdust cultivated was significantly higher (*p* < 0.05) than that of miscellaneous sawdust. However, as major secondary metabolites, polyphenols were involved in defense mechanisms and became excellent antioxidants with the potential to counteract the damage caused by active substances and delay or inhibit oxidation [62,63]. Therefore, it is speculated that *A. auricula* cultivated with walnut sawdust has higher nutritional value.

### 3.6. Comprehensive Evaluation of A. auricula Cultivated in Walnut Sawdust

Correlation analysis of the 14 physiological indexes is shown in Table 4. It was found that Zn was negatively correlated significantly with other indexes (*p* < 0.05), while Fe was positively correlated with polysaccharides at *p* < 0.05 level. Furthermore, Cu was significantly positively correlated with Mg and Mn (*p* < 0.05). Zn was positively correlated with Cu at *p* < 0.05 level. The correlation between each indicator was various, suggesting that the information they reflect was overlapping. It was obvious that the growth of *A. auricula* was a complex process. Using only one index to evaluate the adaptability of *A. auricula* to cultivation substrates was imprecise.

PCA was employed to reduce the overlap among the information reflected by 14 physiological indexes. According to the standard of cumulative contribution ratio ≥85% [64], three principal components were selected (Table 5), and their cumulative contribution ratio was 89.430%, which already covered most of the information. Thus, the first three principal components can be applied to summarize the total information of these traits, which was suitable for the growth of the *A. auricula* and could be comprehensively evaluated. As shown in Table 5, these 14 physiological indexes were transformed into three new independent comprehensive indexes (Z1~Z3). The contributions of these three indexes were 50.617%, 26.671% and 12.143%, respectively. In addition, their eigenvalues were 7.086, 3.734 and 1.700, respectively, in which the first principal component contributed the most to the total genetic rate. Moreover, in the eigenvalues of these three principal components, a higher loading of a variable implied a more significant contribution to the variation. The loading of the Z1 indicated that it has a high contribution of Mn and Cu with a positive loading, whereas total sugar exhibited negative loading. The Z2 mainly showed a high positive loading for single bag production and negative loading for soaked rate and protein. The loading was large for Mg and Zn on the Z3, while the soaked rate had negative loading.

The factor scores of the three Zs were obtained via PCA and were then applied to subordinate function analysis (Table 6). In Z1, the U(X1) of ‘1:3’ was the maximum, with a dependent function value of 1.000, indicating that the cultivation substrate of ‘1:3’ was the most suitable for the growth of *A. auricula* in Z1. On the contrary, ‘3:1’ had the smallest U(X1) of 0.000, indicating that ‘3:1’ was the least suitable for the growth of *A. auricula*. As in Z2 and Z3, ‘3:1’ and ‘0:4’ were the most suitable for the growth of *A. auricula* for their subordinate function value of 1.000, but ‘4:0’ and ‘1:3’ showed the lowest suitable ability for the growth of *A. auricula* since its subordinate function value was 0.000.

Based on the contributions of the first three Zs (50.617, 26.671, 12.143), the weights of the four indexes were 0.566, 0.298, and 0.136, respectively. The D value indicates the level of relative suitability for the growth of the cultivated substrate of *A. auricula*. From Table 6, the cultivation substrate of ‘1:3’ had the maximum D value, suggesting that the cultivation substrate had the highest levels of suitability than others. Moreover, ‘4:0’ had the lowest D value, which was ranked as the lowest level suitable for the growth of *A. auricula*.

## 4. Conclusions

In the study, it was found that the growth cycle, yield and quality of *A. auricula* cultivated with walnut sawdust was better than that cultivated with miscellaneous wood, and the 1:3 substrate (miscellaneous sawdust: walnut sawdust) was the most suitable substrate ratio for the growth of *A. auricula*. This was because walnut sawdust not only had a higher content of phenolic substances and macroscopic elements than miscellaneous sawdust, but it also produced higher extracellular enzyme activity than miscellaneous wood during the growth of basidiocarp. The results showed that compared with miscellaneous wood, walnut sawdust-cultivated *A. auricula* not only had faster mycelium growth rate and basidiocarp growth cycle, but also contained higher nutritional quality and mineral element content. Further analysis showed that compared with other substrates, the 1:3 matrix of miscellaneous sawdust and walnut sawdust not only had shorter mycelium growth rate and basidiocarp growth cycle, but also had the best yield and nutritional quality of cultivated *A. auricula*. Finally, the PCA of the above results further confirmed that the 1:3 substrate (miscellaneous sawdust: walnut sawdust) was the most suitable for the growth of *A. auricula*. In conclusion, the high yield and quality of *A. auricula* were cultivated by waste walnut sawdust, which is not only an environmentally friendly way to reuse walnut waste wood, but can also effectively alleviate the conflict between fungus and forest.

## Figures and Tables

**Table 1:** Nutritional mineral element content in walnut sawdust and miscellaneous sawdust.

Macro mineral element	**Species**	**N (mg/g)**	**P (mg/g)**	**K (mg/g)**	**Ca (mg/g)**	**Mg (mg/g)**
	WS	5.31 ± 0.14 ^a^	0.95 ± 0.04 ^a^	2.50 ± 0.07 ^a^	10.48 ± 0.22 ^a^	1.23 ± 0.04 ^a^
	MS	4.31 ± 0.10 ^b^	0.26 ± 0.01 ^b^	1.38 ± 0.02 ^b^	6.74 ± 0.23 ^b^	0.82 ± 0.02 ^b^
Micro mineral element	**Species**	**Fe (mg/kg)**	**Mn (mg/kg)**	**Cu (mg/kg)**	**Zn (mg/kg)**	**B (mg/kg)**
	WS	678.43 ± 23.83 ^b^	11.16 ± 0.68 ^a^	252.62 ± 3.87 ^a^	28.90 ± 1.07 ^b^	195.23 ± 2.66 ^b^
	MS	800.43 ± 60.96 ^a^	6.50 ± 0.94 ^b^	237.76 ± 6.44 ^b^	59.72 ± 1.93 ^a^	223.32 ± 3.22 ^a^

MS, miscellaneous sawdust; WS, walnut sawdust. Different lowercase letters within each column are significantly different at *p* < 0.05 between treatments.

**Table 2:** Nutritional quality of basidiocarp of *A. auricula* cultivated with different walnut supplemental levels.

MS: WS	Protein/%	Fat/%	Polysaccharide (mg/g)	Total Phenols (mg/100 g)	Total Flavonoids (mg/100 g)	Total Sugar (g/100 g)	Moisture Content/%
4:0	9.82 ±0.05 ^a^	1.08 ± 0.03 ^c^	87.19 ± 1.70 ^c^	131.77 ± 8.73 ^d^	95.55 ± 8.13 ^b^	65.55 ± 2.32 ^a^	89.76 ± 0.12 ^a^
3:1	9.74 ± 0.10 ^a^	1.20 ± 0.03 ^b^	89.13 ± 1.08 ^c^	201.14 ± 8.93 ^a^	78.18 ± 7.29 ^c^	63.30 ± 1.91 ^a^	89.37 ± 0.39 ^a^
2:2	9.65 ± 0.19 ^a^	1.33 ± 0.03 ^a^	95.99 ± 2.60 ^b^	188.93 ± 9.78 ^ab^	117.01 ± 7.53 ^a^	56.18 ± 2.09 ^b^	89.66 ± 0.25 ^a^
1:3	9.76 ± 0.07 ^a^	1.33 ± 0.02 ^a^	99.70 ± 1.73 ^a^	172.44 ± 9.36 ^bc^	90.80 ± 7.48 ^bc^	55.55 ± 1.88 ^b^	88.89 ± 0.07 ^b^
0:4	9.79 ± 0.03 ^a^	1.24 ± 0.05 ^b^	88.73 ± 2.39 ^c^	158.28 ± 8.59 ^c^	103.61 ± 7.85 ^ab^	54.66 ± 2.05 ^b^	89.67 ± 0.22 ^a^

MS, miscellaneous sawdust; WS, walnut sawdust. Different lowercase letters within each column are significantly different at *p* < 0.05 between treatments.

**Table 3:** Mineral nutrition of basidiocarp of *A. auricula* cultivated with different walnut supplemental levels.

MS: WS	Ca (mg/g)	Mg (mg/g)	Fe (mg/kg)	Mn (mg/kg)	Zn (mg/kg)	Cu (mg/kg)
4:0	2.85 ± 0.07 ^b^	1.37 ± 0.03 ^c^	431.77 ± 15.81 ^bc^	9.73 ± 0.47 ^c^	41.56 ± 0.78 ^c^	216.67 ± 1.80 ^bc^
3:1	2.23 ± 0.05 ^c^	1.26 ± 0.03 ^d^	425.52 ± 14.77 ^c^	9.27 ± 0.49 ^c^	48.96 ± 0.81 ^a^	212.50 ± 1.56 ^c^
2:2	2.74 ± 0.10 ^b^	1.41 ± 0.05 ^bc^	465.10 ± 25.07 ^b^	11.23 ± 0.98 ^b^	39.69 ± 0.58 ^c^	221.35 ± 4.77 ^b^
1:3	3.36 ± 0.15 ^a^	1.49 ± 0.06 ^b^	545.94 ± 28.31 ^a^	12.94 ± 0.77 ^a^	37.01 ± 1.15 ^d^	227.22 ± 2.36 ^a^
0:4	3.27 ± 0.10 ^a^	1.65 ± 0.05 ^a^	427.91 ± 12.93 ^c^	11.43 ± 0.77 ^b^	46.21 ± 1.08 ^b^	218.36 ± 1.80 ^b^

MS, miscellaneous sawdust; WS, walnut sawdust. Different lowercase letters within each column are significantly different at *p* < 0.05 between treatments.

**Table 4:** Correlation between different walnut supplemental levels and growth index of *A. auricula*.

Correlation Coefficient	Single Bag Production	Soaked Rate	Protein	Fat	Total Sugar	Polysaccharide	Total Phenols	Total Flavonoids	Ca	Fe	Mg	Mn	Cu	Zn
Single bag production	1													
Soaked rate	−0.471	1												
Protein	−0.523	0.063	1											
Fat	0.793	0.072	−0.702	1										
Total sugar	−0.552	−0.173	0.382	−0.859	1									
Polysaccharide	0.628	0.377	−0.545	0.861	−0.624	1								
Total phenols	0.843	−0.622	−0.762	0.604	−0.242	0.387	1							
Total flavonoids	−0.226	0.584	−0.458	0.369	−0.529	0.228	−0.156	1						
Ca	−0.025	0.611	0.330	0.326	−0.650	0.393	−0.512	0.345	1					
Mg	0.0179	0.31	0.235	0.332	−0.763	0.138	−0.370	0.471	0.868	1				
Fe	0.4924	0.4629	−0.194	0.662	−0.494	0.925 *	0.125	0.016	0.553	0.188	1			
Mn	0.4396	0.4855	−0.151	0.765	−0.850	0.807	−0.007	0.352	0.840	0.679	0.834	1		
Cu	0.3137	0.6695	−0.196	0.694	−0.684	0.878	−0.0703	0.369	0.766	0.482	0.917 *	0.947 *	1	
Zn	0.0254	−0.8754	0.193	−0.421	0.329	−0.759	0.2408	−0.420	−0.564	−0.179	−0.808	−0.670	−0.882 *	1

The data are the average of the correlation coefficient correlation of *A. auricula* with different walnut addition levels. * means significant at the level of 0.05.

**Table 5:** Correlation between different walnut supplemental levels and growth index of *A. auricula*.

Index		Z1	Z2	Z3
Eigenvalues		7.086	3.734	1.700
Contribution ratio/%		50.617	26.671	12.143
Cumulative contribution ratio/%		50.617	77.287	89.430
Eigenvector	Single bag production	0.167	0.426	0.154
	Soaked rate	0.206	−0.354	−0.326
	Protein	−0.133	−0.363	0.172
	Fat	0.313	0.271	0.096
	Total sugar	−0.313	−0.080	−0.387
	Polysaccharide	0.335	0.165	−0.232
	Total phenols	0.335	0.165	−0.232
	Total flavonoids	0.177	−0.130	0.061
	Ca	0.274	−0.296	0.246
	Mg	0.208	−0.223	0.533
	Fe	0.320	0.039	−0.249
	Mn	0.364	−0.044	0.123
	Cu	0.363	−0.077	−0.120
	Zn	−0.292	0.160	0.419

**Table 6:** The factor scores of comprehensive indexes, index weight, U(Xj) and D value of *A. auricula* cultivated at different walnut addition levels.

MS:WS	Z1	Z2	Z3	U(X1)	U(X2)	U(X3)	D	Rank
4:0	243.489	71.249	−151.019	0.083	0.000	0.777	0.172	5
3:1	238.782	113.547	−145.267	0.000	1.000	0.931	0.441	3
2:2	269.266	102.425	−156.569	0.539	0.737	0.630	0.611	2
1:3	295.305	100.569	−180.233	1.000	0.693	0.000	0.749	1
0:4	249.094	86.704	−142.658	0.182	0.365	1.000	0.368	4
Index weight	50.617	26.671	12.143	0.566	0.298	0.136		

Note: MS: miscellaneous sawdust; WS, walnut sawdust.

## Data Availability

Not applicable.

## References

[1] Zhao Y., Wang L., Zhang D., Li R., Cheng T., Zhang Y., Liu X., Wong G., Tang Y., Wang H. (2019). Comparative transcription analysis reveals relationship of three major domesticated varieties of *Auricularia auricula*-judae. Sci. Rep..

[2] Ren Y.C., Huang J.T., Wang X.N., Wang Y.Q., Li H.C., Yue T.L. (2022). Effect of sulfite treatment on the quality of black fungus. Food Chem..

[3] Zhao Q., Sulayman M., Zhu X.T., Zhao Y.C., Yang Z.L., Hyde K.D. (2016). Species clarification of the culinary *Bachu* mushroom in western China. Mycologia.

[4] Zheng H.C. (2017). Quality management measures for the production of *Auricularia auricula*-judae edible fungus. Int. J. Med. Mushrooms.

[5] Sun S., Zhang X., Chen X., Zhang L., Zhu H. (2016). Production of natural edible melanin by *Auricularia auricula* and its physicochemical properties. Food Chem..

[6] Li L., Fan X.Z., Xiao Y., Zhou Y., Bian Y.B. (2010). The physiological characteristics and genetic diversity analysis of *Auricularia auricula*-judae cultivated germplasm in China. Mycosystema.

[7] Chen S.Y., Wu Q.P., Zhou X.Y., Que S.H., Qiu W.Y. (2002). Research progress on cultivation of edible fungi with coniferous sawdust. J. Microbiol..

[8] Zhang J.P., Du M.H. (2017). Application status and prospect of *Pinus massoniana* sawdust as the substrate for the cultivation of Edible fungi. Food Nutr. Sci..

[9] Liu B.H., Zhao D.C., Zhang P.Y., Liu F.C., Jia M., Liang J. (2020). Seedling evaluation of six walnut rootstock species originated in China based on principal component analysis and cluster analysis. Sci. Hortic..

[10] Rugolo M., Lechner B., Mansilla R., Mata G., Rajchenberg M. (2020). Evaluation of *PleurotusostreatusBasidiomes* production on Pinus sawdust and other agricultural and forestry wastes from Patagonia, Argrntina. Maderas-Ciens Technol..

[11] Galic M., Stajic M., Vukojevic J., Cilerdzic J. (2020). Capacity of *Auricularia auricula*-judae to degrade agroforestry residues. Cellul. Chem. Technol..

[12] Manavalan T., Manavalan A., Heese K. (2015). Characterization of lignocellulolytic enzymes from white-rot fungi. Curr. Microbiol..

[13] Liu Z.B., Xu S., Sun X.Z., Li Y., Song B. (2021). Quality of Lentinus edodes cultivated with corn straw and safety evaluation of heavy metal elements. Chin. Edible Fungi.

[14] Lakhtar H., Ismaili-Alaoui M., Philippoussis A., Perraud-Gaime I., Roussos S. (2010). Screening of strains of Lentinula edodes grown on model olive mill wastewater in solid and liquid state culture for polyphenol biodegradation. Int. Biodeter. Biodegr..

[15] Ntougias S., Baldrian P., Ehaliotis C., Nerud F., Antoniou T., Merhautova V. (2012). Biodegradation and detoxification of olive mill wastewater by selected strains of the mushroom genera Ganoderma and *Pleurotus*. Chemosphere.

[16] Zervakis G., Yiatras P., Balis C. (1996). Edible mushrooms from olive mill wastes. Int. Biodeter. Biodegr..

[17] Philippoussis A., Diamantopoulou P., Papadopoulou K., Lakhtar H., Roussos S., Parissopoulos G., Papanikolaou S. (2011). Biomass, laccase and endoglucanase production by Lentinula edodes during soild-state fermentation of reed grass, bean stalks and wheat straw residues. World J. Microbiol. Biotechnol..

[18] Hu X.Z. (2011). Research progress of lignin degrading enzyme. J. Anhui Agric. Sci..

[19] Sun Y.Z., Yang L.X., Wang D.L. (2013). Relationship between walnut quinone and soil microbe population in Manchuria juglans forest. Chin. J. Appl. Ecol..

[20] Maria V.O., Raul F.F., Tomas G.J., Oscar A.C., Juan A., Anabela M.A. (2018). Use of lignocellulosic wastes of pecan (*Carya illinoinensis*) in the cultivation of Ganoderma lucidum. Rev. Micol..

[21] Hao Z.K., Zhang W.E., Tian F.H., Wei R., Fu L.R., Pan X.J. (2022). Comprehensive evaluation of the adaptability of 11 edible mushroom in *Juglans sigillata* Dode sawdust substrate. Seed.

[22] Lu M., Yan Q.L., Chi Y., Zheng H.Y., Xiao L.J. (2016). Production of liquid spawn of an edible mushroom, *Sparassis latifolia* by submerged fermentation and mycelial growth on pine wood sawdust. Sci. Hort..

[23] Singh U., Gautam A., Singha T.K., Tiwari A., Tiwari P., Sahai V., Sharma S. (2020). Mass production of *Pleurotuseryngii* mycelia under submerged culture conditions with improved minerals and vitamin D2. LWT.

[24] Cheng Y.C., Li J.H., Su C.Z., Zhang Z.H., Hu K.H. (2020). Effects of temperature on the physiological activity of Mushroom at post-ripening stage. Edible Fungi China.

[25] Wang M. (2019). Discussion on the method of determination of organic carbon content in soil by dimitric acid bell oxidation and external heating. Xinjiang YouseJinshu.

[26] Yan X.Z., Liu T.J., Zhao H.Y. (2020). The protein content in donkey-hide products is determined by the Keshi method. China Food Saf. Mag..

[27] Wang J.L., Han G., Yan X.J., Luo J.Q., Du X., Dong H.Y. (2017). Determination of seven elements in artificial diamond by inductively coupled plasma atomic emission spectrometry. Metall. Anal..

[28] Wang L.J., Rao F.J., Sun X., Chen H.T., Qin H.Y., Zhan L.X. (2022). Effect of transtubular succession on quality of *Auricularia heimuer* spawns. J. Jilin Agric. Univ..

[29] Liu J.N., Dang A.L., Zhang P.Q., Dai X.D., Zhang J.C. (2012). Purification of Laccase from *Auricularia auriculata* and Characterization of part of Laccase. Fungus Res..

[30] Yuan B., Zhou S.Y., Liu C.W., Zhang S., Yan P., Liu A.L. (2020). Study on extraction technology and activity determination of polyphenol oxidase. Tea Commun..

[31] An Q., Wu X.J., Wu B., Dai Y.C. (2015). Effects of different carbon and nitrogen sources on lignocellulosic activity of *Flammulinavelutipes*. Mycosystema.

[32] Li J.J., Jiang Y.C., Meng Q.X., Dan Q.K., Wang Y., Zhu S.W., Hua X.P. (2018). Optimum environment parameters of the growth of *Auricularia auricula* HW15. Edible Fungi China.

[33] Barros L., Venturini B.A., Baptista P., Estevinho L.M., Ferreira I.C. (2008). Chemical composition and biological properties of Portuguese wild mushrooms, a comprehensive study. J. Agric Food Chem..

[34] Song J.L., Yuan W.D., Zhou Z.F., Wang W.K., Lu N., Cheng J.W., Yan J. (2020). Activities of extracellular enzymes and polysaccharides in liquid culture of *Sanghuangporus*. Mycosystema.

[35] Wang C.L., Zhang w.e., Pan X.J. (2015). Study on extraction process of Total Polyphenols from male inflorescences of Walnut. Food Ind..

[36] Wang S., Wang Z., Wang P. (2011). Evaluation of wheat freezing resistance based on the responses of the physiological indices to low temperature stress. Acta Ecol..

[37] Zhou G.S., Mei F.Z., Zhou Z.Q., Zhu X.T. (2003). Comprehensive evaluation of moisture tolerance of different wheat varieties (lines). J. Biomath..

[38] Gong Z.G., Wang Y.F., Wang H., Li W., Geng M.J., Zhang W.M., Liu L. (2021). Research progress on mineral nutrition of walnut. Sci. Silvae Sin..

[39] Dean H., Colby (2001). Plant Food Elements Withdrawn from the Soil by Fruits II California Walnut Growers Association. The California Walnut.

[40] Srivastava A.K., Hu C.X. (2014). Nutrient Management in Fruit Crops: Issues and Strategies. Indian J. Fertil..

[41] Stajić M., Persky L., Friesem D., Hadar Y., Wasser S.P., Nevo E., Vukojević J. (2006). Effect of different carbon and nitrogen sources on laccase and peroxidases production by selected *Pleurotus* species. Enzym. Microb. Tech..

[42] Osma J.F., Saravia V., Herrera J.L.T., Couto S.R. (2007). Mandarin peelings: The best carbon source to produce laccase by static cultures of *Trametespubescens*. Chemosphere.

[43] Li P., Wang H., Liu G., Li X., Yao J. (2011). The effect of carbon source succession on laccase activity in the co-culture process of Ganoderma lucidum and a yeast. Enzym. Microb. Technol..

[44] Adil B., Xiang Q.J., He M.L., Wu Y.T., Asghar M.A., Arshad M., Qin P., Gu Y.F., Yu X.M., Zhao K. (2020). Effect of sodium and calcium on polysaccharide production and the activities of enzymes involved in the polysaccharide synthesis of Lentinus edodes. AMB Expr..

[45] Han Z.H., Zhang P.Q., Kong X.H., Ma Q.F., Dai X.D., Zhang J.C. (2007). Study on changes of extracellular enzyme activity in *Auricularia auriculata* and comparison of cultivated characters. Chin. J. Edible Fungi.

[46] Wang Y.J., Fu L.Z., Han Z.H., Dai X.D., Kong X.H., Zhang J.C. (2011). Research progress of the extracellular enzyme of *Auricularia auricular*. Heilongjiang Sci..

[47] Fang H.Y., Ren Z.M., Meng X.X., Dai J.J., Li S.J., Li Z.W., Li X. (2018). The relationship between extracellular enzymes changes and agronomic traits in *Auricularia fuscosuccinea*. Mol. Plant Breed..

[48] Alcalde M., Polaina J., MacCabe A.P. (2007). Laccases: Biological Functions, Molecular Structure and Industrial Applications. Industrial Enzyms.

[49] Si J., Cui B.K., He S., Dai Y.C. (2011). Optimization of conditions for laccase production by *Perenniporiasubacida* and its application in dye decolorization. Chin. J. Appl. Environ. Biol..

[50] Wu Y., Ma H.F., Cao Y.J., Si J., Cui B.K. (2019). Advances on properties, production, purification and immobilization of fungal laccase. Biotechnol. Bull..

[51] Wu Z.W. (2012). Study on the Effects of Cultivation Formula, Cu^(2+)^ and Mn^(2+)^ on The Growth and Development of Pleurotuscylindracea. Master’s Thesis.

[52] Zhu C.W., Bao G.W., Huang S. (2015). *Pleurotusostreatus* laccase for different heavy metals stress response. J. Environ. Sci. Res..

[53] Pandey R.K., Tewari S., Tewari L. (2018). *Lignolytic* mushroom *Lenzites* elegans WDP2: Laccase production, characterization, and bioremediation of synthetic dyes. Ecotoxicol. Environ. Saf..

[54] Xu X.H., Chen A., Pei Y., Wu L.J., Wen Y., Zhang W.P. (2021). Analysis of Extracellular Enzymes Activity of Liquid and Solid Strains of *Lentinus edodes* in Different Media. Mol. Plant Breed..

[55] He C.L., Li M., Tian J.H., Dong Y.N., Li S.M. (2019). Study on Effective Cultivation Formula Selection and Extracellular Enzymes Activity of *Lentinus edodes* with Grape Sawdust. Edible Fungi China.

[56] Zhao C.M., Du F., Zou Y.J., Hu Q.X., Zheng S.Y. (2020). Study on the relationship between mycelial growth and lignin degrading enzymes of *Pleurotuseryngii* based on different substrates. Edible Fungi China.

[57] Rani P., Kalyani N., Prathiba K. (2008). Evaluation of *Lignocellulosci* wastes for production of Edible Mushrooms. Appl. Biochem..

[58] Tanesaka E., Takeda H., Yoshida M. (2013). Phenol-Oxidizing enzyme expression in Lentinula edodes by the addition of sawdust extract, aromatic compounds, or copper in liquid culture media. Biocontrol Sci..

[59] Isikhuemhen O.S., Mikiashvilli N.A. (2009). Lignocellulolytic enzyme activity, substrate utilization, and mushroom yield by *Pleurotusostreatus* cultivated on substrate containing anaerobic digester solids. J. Ind. Microbiol. Biotechnol..

[60] Koutrotsios G., Tagkouli D., Bekiaris G., Kaliora A., Tsiaka T., Tsiantas K., Chatzipavlidis I., Zoumpoulakis P., Kalogeropoulos N., Zervakis G.I. (2022). Enhancing the nutritional and functional properties of *Pleurotuscitrinopileatus* mushrooms through the exploitation of winery and olive mill wastes. Food Chem..

[61] Basso V., Schiavenin C., Mendonca S., Goncalves F., Salvador M., Camassola M. (2020). Chemical features and antioxidant profile by *Schizophyllun commune* produced on different agroindustrial wastes and byproducts of biodiesel production. Food Chem..

[62] Dubost N.J., Ou B., Beelman R.B. (2007). Quantification of polyphenols and ergothioneine in cultivated mushrooms and correlation to total antioxidant capacity. Food Chem..

[63] Gambato G., Todescato K., Paväo E.M., Scortegagna A., Fontana R.C., Salvador M., Camassola M. (2016). Evaluation of productivity and antioxidant profile of solid-state cultivated macrofungi *Pleurotusalbidus* and *Pycnoporussanguineus*. Bioresour. Technol..

[64] Tang Q.Y., Feng M.G. (2002). Practical Statistical Analysis and Its DPS Data Processing System.

